# Validity of Smartphone Heart Rate Variability Pre- and Post-Resistance Exercise

**DOI:** 10.3390/s20205738

**Published:** 2020-10-09

**Authors:** Clifton J. Holmes, Michael V. Fedewa, Lee J. Winchester, Hayley V. MacDonald, Stefanie A. Wind, Michael R. Esco

**Affiliations:** 1 Program in Physical Therapy, Washington University School of Medicine, St. Louis, MO 63110, USA; 2 Department of Kinesiology, University of Alabama, Tuscaloosa, AL 35487, USA; mvfedewa@ua.edu (M.V.F.); ljwinchester@ua.edu (L.J.W.); hvmacdonald@ua.edu (H.V.M.); stefanie.wind@ua.edu (S.A.W.); mresco@ua.edu (M.R.E.)

**Keywords:** photoplethysmography, autonomic modulation, pulse rate variability, mobile devices, fatigue, recovery

## Abstract

The aim was to examine the validity of heart rate variability (HRV) measurements from photoplethysmography (PPG) via a smartphone application pre- and post-resistance exercise (RE) and to examine the intraday and interday reliability of the smartphone PPG method. Thirty-one adults underwent two simultaneous ultrashort-term electrocardiograph (ECG) and PPG measurements followed by 1-repetition maximum testing for back squats, bench presses, and bent-over rows. The participants then performed RE, where simultaneous ultrashort-term ECG and PPG measurements were taken: two pre- and one post-exercise. The natural logarithm of the root mean square of successive normal-to-normal (R-R) differences (LnRMSSD) values were compared with paired-sample *t*-tests, Pearson product correlations, Cohen’s *d* effect sizes (ESs), and Bland–Altman analysis. Intra-class correlations (ICC) were determined between PPG LnRMSSDs. Significant, small–moderate differences were found for all measurements between ECG and PPG: Base_Pre1_ (ES = 0.42), Base_Pre2_ (0.30), RE_Pre1_ (0.26), RE_Pre2_ (0.36), and RE_Post_ (1.14). The correlations ranged from moderate to very large: Base_Pre1_ (*r* = 0.59), Base_Pre2_ (*r* = 0.63), RE_Pre1_ (*r* = 0.63), RE_Pre2_ (*r* = 0.76), and RE_Post_ (*r* = 0.41)—all *p* < 0.05. The agreement for all the measurements was “moderate” (0.10–0.16). The PPG LnRMSSD exhibited “nearly-perfect” intraday reliability (ICC = 0.91) and “very large” interday reliability (0.88). The smartphone PPG was comparable to the ECG for measuring HRV at rest, but with larger error after resistance exercise.

## 1. Introduction

Advances in technology have produced a plethora of commercially available mobile devices that can measure health and fitness outcomes. These mobile devices have seen an expansive increase in use in medicine, healthcare and the military but most notably through personal use by athletes and active individuals of the general population. According to the American College of Sports Medicine (ACSM), wearable technology has remained in the top three fitness trends since 2016 [1]. Though physical activity monitors are widely used to measure daily step counts and estimate energy expenditure, both fitness enthusiasts and recreationally trained athletes alike seek tools for gauging recovery and readiness to perform. Of the new methods and equipment currently available, heart rate variability (HRV) assessment has emerged as one of the more useful and practically applied tools.

Heart rate variability is defined as the oscillations that occur between successive heartbeats and is considered a non-invasive marker of autonomic nervous system (ANS) control of the cardiovascular system [2]. The assessment of HRV has been utilized by athletes to indirectly examine ANS status in response to various types of training [3,4] and has been demonstrated as a valid tool for monitoring fatigue accumulation and recovery [5,6]. The majority of HRV research occurs in controlled laboratory settings with sophisticated equipment, such as an electrocardiograms (ECGs) [7]. The criterion method of ECG for acquiring HRV data requires 10 min short-term measures consisting of a 5 min stabilization period followed by a 5 min recording period [2]. Unfortunately, these methods are ill-suited for field conditions, especially when daily measures are recommended [6].

Technological advances have allowed HRV to be collected with mobile devices. A recent meta-analysis consisting of twenty-three studies concluded that portable devices yielded small but acceptable ranges of error in comparison to ECG (effect size (ES) = 0.23, 95% CI: 0.05, 0.42) [7]. Photoplethysmography (PPG) has been proposed as a surrogate for traditional ECG for the measurement of HRV [8]. The PPG method is a noninvasive optical technique for monitoring beat-to-beat relative blood volume changes in the microvasculature of peripheral tissues. The pulse rate variability (PRV) of the PPG signal has been highly correlated with both time and frequency-domain metrics from ECG-derived HRV indices [9,10]. Several PPG-based HRV smartphone applications have been validated in the literature [7,11,12,13] for acquiring the parasympathetically derived marker of the root mean square of successive R–R interval differences (RMSSD) [11,14,15]. Previous research has suggested that the RMSSD is the preferred HRV metric for field recordings, primarily because it can be accurately measured with an ultra-shortened recording time of only one min [14] following a 1 min stabilization period [16].

It is recommended to measure HRV at least three days per week as a minimum requirement to account for common daily fluctuations [4,17]. The frequent measurement of HRV has recently become more feasible because of the emerging smartphone PPG technology mentioned above. However, several concerns related to the agreement between mobile PPG smartphone applications and ECG for HRV determination exist. For instance, physical and mental stimuli have been suggested to decrease the agreement between PPG and ECG [10]. Furthermore, only a few studies have validated smartphone PPG technology for measuring HRV, and there is no existing research to determine the reliability of such an approach across a period of days or following physical exercise. This is a significant gap in the literature, considering the recommendation of daily HRV assessment in athletes and the stressors received from frequent training. Therefore, the present study aimed to examine the validity of HRV measures from a PPG smartphone application under resting and post-resistance exercise conditions. The secondary aim was to examine the intraday and interday reliability of the PPG method. It was hypothesized that no significant mean differences and large correlations would be shown between the ECG and PPG smartphone application at all-time points, and that the application would demonstrate “near perfect” levels of intra- and interday reliability. 

## 2. Materials and Methods

### 2.1. Participants

Thirty-one healthy, resistance-trained adults (29.0% female, age: 23.9 ± 5.4 y, height: 171.0 ± 21.5 cm, mass: 75.9 ± 12.9 kg, body fat: 17.6 ± 7.2%, and experience: 5.1 ± 1.1 y) participated in this study. The participants’ self-reported demographics included 24 white/Caucasians, 5 black/African-Americans, 1 Asian, and 1 Hispanic/Latino. The participants were actively engaged in resistance exercise at least three days per week for 3 months leading up to the start of data collection. The participants were free from the presence of symptoms of cardiovascular, metabolic, musculoskeletal and renal disorders or diseases. This project was approved by the university‘s Institutional Review Board and conformed to the Declaration of Helsinki.

### 2.2. Experimental Design

The study required the participants to visit the lab three times over approximately two weeks. All the testing sessions began between 6:00 and 11:00 a.m. to control diurnal variations, and the participants reported to the lab within the same 2-hour period for every session. During the initial visit, participants provided written informed consent, completed screening questionnaires, and were familiarized with all the testing procedures. During the second visit, two simultaneous ultrashort-term ECG and PPG measurements of HRV were taken, with blood pressure assessment occurring between the recordings. After HRV assessment, 1-repetition maximum (1RM) testing for the Back Squat (BS), Bench Press (BP), and Bent-over Row (BR) exercises was performed. During the third visit, the participants performed a hypertrophic-style, full-body resistance exercise protocol. Two simultaneous HRV recordings were performed pre-exercise, and one measurement was performed 5 min post-exercise.

### 2.3. Baseline Session

Upon arrival at the lab, anthropometrics and body composition measurements were taken during the second visit. Participants were asked to come to the lab having refrained from eating any heavy meals (≤300 calories) or drinking beverages other than water (≤500 mL) two hours before arrival. Nude body mass was measured (to the nearest 0.1 kg) with a calibrated digital scale (Tanita BWB-800, Tanita©, Arlington Heights, IL, USA), and standing height was measured (to the nearest 0.1 cm) with a stadiometer (SECA 213, Seca Ltd., Hamburg, Germany). Body composition (%Fat) was measured via skinfold (SKF) thickness for descriptive purposes, with two measurements (within 2 mm of each other) taken using calibrated skinfold calipers (Lange Skinfold Caliper, Seko, USA) across 7 standard sites on the right side of the body. The %Fat was estimated using the Brozek equation (%Fat = ((4.57/BD) − 4.142) × 100) [18]. 

Following the completion of SKF testing, HRV was assessed in the seated position using both criterion ECG methods and a previously validated [13] smartphone application, HRV4Training (see https://www.hrv4training.com/). The HRV4Training application utilizes a configuration of PPG where the blood-perfused tissue (i.e., fingertip) is placed over the source of light (i.e., flash) and the detector (i.e., camera) simultaneously [10]. The camera records small variations in light absorption as the beat-by-beat capillary blood volume fluctuates between successive systolic and diastolic cardiac cycles [19]. The ECG signals were collected with an electronic signal acquisition system (BIOPAC MP150 Physiograph) connected to a Dell PC. The Acknowledge software (v 4.4, BIOPAC, Goletta, CA, USA) was used to collect real-time ECGs. The ECG assessment was performed with a modified lead II configuration, where three surface electrodes (BIOPAC EL504 disposable Ag-AgCl) were placed at the following anatomical locations: (1) negative polarity over the right midclavicular notch, (2) positive polarity over the fifth intercostal space along the midaxillary line, and (3) ground placement over the iliac crest of the left hip along the midclavicular line. All the HRV recordings took place in a quiet room maintained at a temperature of 20–23 °C. The heart rate variability measurements consisted of a 1 min recording timeframe following a 5 min stabilization period, with spontaneous breathing patterns throughout [20]. 

The reliability of the PPG measurements was assessed according to two components: intraday, comparing measurements collected within the same day (i.e., baseline session), and interday, comparing measurements collected on two separate days (i.e., baseline and exercise session) [21]. Reliability was only assessed between measurements taken under resting conditions to avoid measurement discrepancies due to vast differences in physiological states pre- and post-resistance exercise (RE). The recordings took place at the following time points: twice during the baseline visit (Base_Pre1_ and Base_Pre2_); twice before (RE_Pre1_ and RE_Pre2_) and once 5 min after (RE_Post_) the high-volume resistance exercise trial. Successive recordings during a specific period (Base_Pre1_ and Base_Pre2_; RE_Pre1_ and RE_Pre2_) were separated by 10 min of quiet rest. Each recording commenced when the tip of the right index finger was positioned to cover the camera and flash of the smartphone (iPhone 6, Apple Inc., Foxconn, Pegatron, Taipei, Taiwan). A technician manually marked the ECG within the Acknowledge software at the precise commencement and completion of the 1 min PPG recording [11,13]. For the consistency of the measurement, the same smartphone device was used for all measurements. 

Blood pressure changes have been shown to affect PRV, thus leading to discrepancies in HRV measurements [22,23]. For this reason, additional blood pressure assessments, outside of screening purposes, were performed to aid in explaining any possible differences seen in HRV between the ECG and PPG. Assessments were conducted with the BPM-100 automated blood pressure monitor (BpTRU Medical Devices; Coquitlam, Canada) at least three times, 2 min apart, in the dominant arm [24]. In addition to assessing systolic and diastolic blood pressure changes, the pulse pressure (PP) (systolic–diastolic) and mean arterial pressure (diastolic + 0.333 × PP) were calculated. 

The RMSSD metric was solely investigated due to its previous validation with ultrashort-term measures [14] and with the current smartphone application [13]. All ECG segments were transformed into a tachogram using the Acknowledge software and exported into the Kubios HRV Standard 3.3.0 software (Biosignal Analysis Medical Imaging Group at the Department of Applied Physics, University of Kuopio, Kuopio, Finland). Occasional artifact noise was automatically replaced with the interpolated adjacent RR interval values (threshold = 0.45 s or “very low”), amounting to ≤3% of error correction. The analysis process was carried out by the same researcher to ensure consistency [21,25]. For the PPG measurements, erroneous data were discarded from any recording. The data-capturing application was designed to inform the user whether data were of sufficient quality or not. The automated and proprietary algorithm of the application identifies periods of high noise by analyzing the percentage of discarded RR intervals over a given time [13]. High-noise periods were based on when timing differences were outside of the expected or normal values, typically due to underlying noise or ectopic beats [13]. In cases where the RMSSD data attained were inappropriate due to participant error (e.g., the movement of the finger over the camera), the participant was informed and the ECG data were discarded. A total of up to three measurements were taken to achieve a signal quality of “good” to “optimal”; otherwise, data from the third measurement were saved for analysis. Measurements were taken consecutively without additional stabilization periods, and the participants were given instructions to improve the signal quality as provided by the HRVTraining website (see https://www.hrv4training.com/). To ensure measurement quality, the participants were instructed to remain as still as possible, refrain from speaking, and use a “light touch” without an overabundance of contact pressure with the index fingertip when performing measurements. Attempts were limited to three since additional measurements with smartphone PPG would eclipse the traditional 5 min recording periods, thus eliminating the practicality of ultrashort-term measures. After the collection of HRV and blood pressure, the participants completed 1RM testing for BS, BP, and BR using procedures adapted from previously reported protocols [26]. 

### 2.4. Resistance Exercise Session 

At least 72 h after the 1RM testing was completed, the participants performed a full-body bout of resistance exercise. Upon completion of the warm-up, the participants completed the resistance exercise protocol consisting of 6 sets of 10 repetitions of BSs, 3 sets of 10 repetitions of BPs, and 3 sets of 10 repetitions of BRs. The relative load was 70% of 1RM for all the exercises, with 120 s of rest between each set and 180 s of rest between each exercise [27,28]. If a participant reached failure during a set before completing the prescribed 10 repetitions, 30–60 s of rest was allowed before continuing with the set. This process was repeated until 10 repetitions were completed. 

### 2.5. Statistical Analysis

All data were analyzed with IBM SPSS version 25.0 for Windows (Somers, NY) and Microsoft Excel 2016 for Windows (Microsoft Corporation, Redmond, WA). Non-normal (i.e., skewed) distributions are common among the collected HRV group data [14,17]. To prevent this and ensure a normal distribution for the collected data, the natural logarithmic transformation of the RMSSD (LnRMSSD) was applied [29,30]. The mean values of the PPG and criterion ECG LnRMSSD measurements were compared with paired-sample *t*-tests. The magnitude of the difference for each pair-wise comparison was quantified using Cohen’s *d* effect size (ES) and classified as trivial (0.0–0.2), small (0.2–0.6), moderate (0.6–1.2), large (1.2–2.0), or very large (>2.0) [31,32]. Pearson product-moment correlation coefficients (*r*) were calculated to assess the association between the ECG and PPG-derived LnRMSSD values. Agreement between the ultrashort-term LnRMSSD values was evaluated using Bland–Altman analysis. The agreement was quantified as the calculated ratio of half the 95% confidence interval (CI) and the mean of the average values, where a “good” agreement was considered if the ratio was less than 0.1, “moderate” agreement was considered if the ratio was 0.1–0.2, and “insufficient” agreement, if the ratio was >0.2 [10,15]. The validation statistics also involved calculating the standard error of the estimate (SEE) for the PPG values against ECG. 

Intraday reliability was determined by comparing the PPG measurements from Base_Pre1_ and Base_Pre2_. Interday reliability was determined by comparing the PPG measurements from Base_Pre1_, Base_Pre2_, RE_Pre1_, and RE_Pre2_. Reliability statistics were calculated using Friedman chi-square (χ**^2^**), and intra-class correlations (ICC) were determined between the LnRMSSD parameters. Correlation values 0 to 0.30 were considered small, 0.31 to 0.49 was moderate, 0.50 to 0.69 was large, 0.70 to 0.89 was very large, and 0.90 to 1.00 was near perfect [32]. Repeated-measures analysis of variance (ANOVA) with Bonferroni correction was performed to assess differences in pre- and post-exercise heart rates (HRs) and blood pressure metrics (e.g., systolic, diastolic, pulse pressure [PP], mean arterial pressure [MAP]). The partial eta-squared (η^2^) of the repeated-measures ANOVA was used to determine the ES for HR and blood pressure differences. Unless otherwise stated, all data are presented as mean ± standard deviation (*M* ± *SD*), and statistical significance was accepted at *p* < 0.05.

## 3. Results

### 3.1. Validity of Photoplethysmography

**Figure 1:**
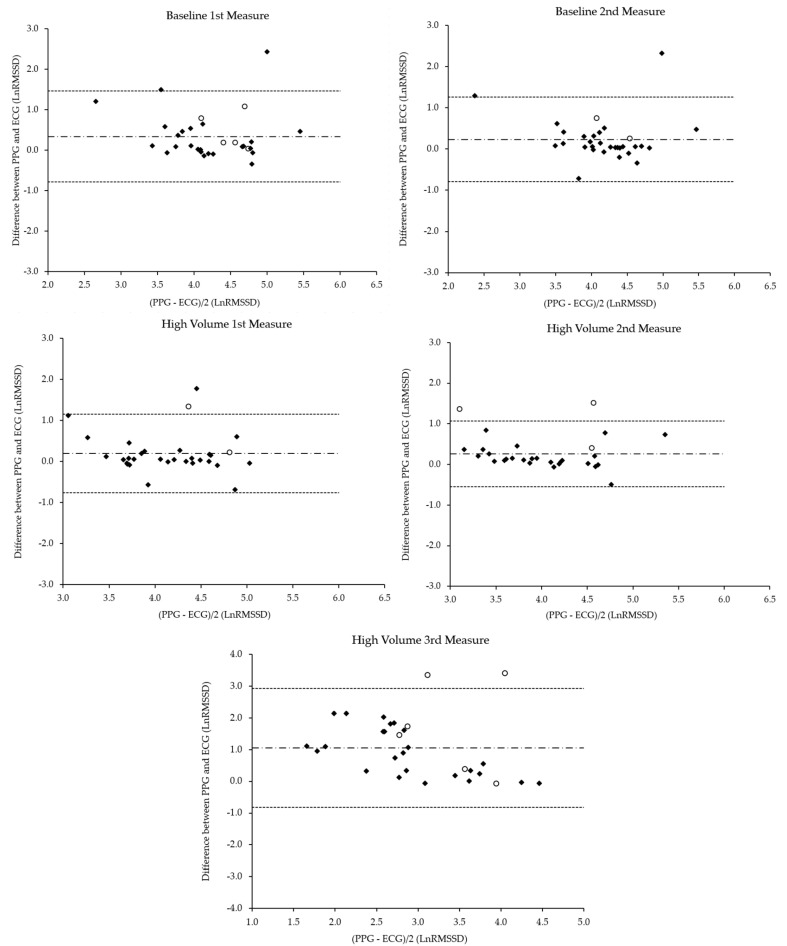
Bland–Altman plots comparing the log transformation of the root mean square of successive RR differences (LnRMSSD) values from the smartphone application photoplethysmography (PPG) with the criterion electrocardiogram (ECG). The solid lines represent the mean bias, whereas the outside dashed lines represent the 95% limits of agreement. Black diamonds represent “optimal” and “good” signal quality measures, and white circles represent “failed” signal quality.

The LnRMSSD values of both the PPG and ECG as well as validity statistics are displayed in Table 1. Paired-sample *t*-tests showed statistically significant differences (*p* <0.05) for all the resting comparison measurements of ECG and PPG under resting conditions. However, all the Cohen’s *d* effect sizes were quantitatively classified as small. Significant correlations ranging from large to very large were also observed for the pre-exercise measurements. The constant error (CE) and SEE values were consistent with all the resting measurements, with the CE values falling within the upper and lower ranges of the limits of agreement. The quality of the agreement for all the pre-exercise measurements, according to the Bland–Altman ratio, was classified as “good”. The plots can be seen in Figure 1. A moderate, statistically significant mean difference was observed between the ECG and PGG measurements post-exercise. However, a moderate correlation was found between the post-exercise recordings, relatively weaker than that under resting conditions. The quality of agreement was determined to be “moderate”, with SEE values increasing from pre-exercise recordings but CE remaining within the upper and lower ranges of the limits of agreement. 

For further analysis of the validity, the signal quality of all the PPG measurements was recorded, resulting in a total of 153 measurements with 66.7% “optimal”, 21.6% “good”, and 11.8% “not optimal”. The paired-sample t-tests and correlation results for each signal quality category can be seen in Table 2. The Bland–Altman ratios for each category followed a similar trend, with all baseline and pre-exercise measurements displaying “good” agreement and all post-exercise measurements being “insufficient”.

### 3.2. Reliability of Photoplethysmography

All the intraday and interday reliability statistics are displayed in Table 3. The PPG-derived LnRMSSD baseline measurements, for intraday reliability, had non-significant Friedman chi-square values (χ^2^ = 2.08, *p* = 0.149), while the ICC values were “nearly perfect” (ICC = 0.91). For interday reliability, four measurements were compared: Base_Pre1_, Base_Pre2_, RE_Pre1_, and RE_Pre2_. The chi-square value was also not statistically significant (χ^2^ = 5.02, *p* = 0.171), with an ICC “very large” (ICC = 0.88). 

### 3.3. Resting Heart Rate and Blood Pressure

The overall *F* for the differences in the mean HR measurements at baseline (67.44 ± 13.15), pre-exercise (66.31 ± 9.78), and post-exercise (94.46 ± 12.90) were statistically significant: *F* (2, 58) = 100.113, *p* < 0.001. The corresponding estimated ES was a partial η^2^ of 0.775. The post-exercise HR was significantly higher than baseline (*p* < 0.001) and pre-exercise levels (*p* < 0.001). No significant differences were observed in the systolic blood pressure values (*p* = 0.640). 

Diastolic measures at baseline (70.14 ± 6.73 mmHg), pre-exercise (69.66 ± 7.86 mmHg), and post-exercise (64.91 ± 6.59 mmHg) were statistically significant: *F* (2, 60) = 6.496, *p* = 0.003. The corresponding estimated ES was a partial η^2^ of 0.178. With an adjusted *p*-value (0.05/3) of 0.02, the post-exercise diastolic blood pressure was significantly lower than baseline (*p* = 0.02) and pre-exercise levels (*p* = 0.016). The pulse pressure values at baseline (34.35 ± 14.79 mmHg), pre-exercise (36.13 ± 11.14 mmHg), and post-exercise (42.53 ± 11.28 mmHg) were statistically significant: *F* (1.65, 49.36) = 4.149, *p* = 0.028. The estimated ES was a partial η^2^ of 0.122. The post-exercise PP was significantly higher than pre-exercise PP (*p* = 0.02). No significant differences were observed in the MAP values (*p* = 0.297).

## 4. Discussion

Under resting conditions, significant differences were found between all simultaneous ECG and PPG measures, yet the effect sizes were considered small with strong correlations and moderate agreement between methods. Post-exercise simultaneous HRV measures were also significantly different; however, the two methods exhibited a moderate ES difference and the lowest correlation, yet presented moderate agreement. Finally, strong reliability was displayed by smartphone PPG measurements within days and between days. These findings demonstrate that smartphone PPG is a valid and reliable surrogate that can be utilized daily, but significantly overestimates LnRMSSD following resistance exercise. 

Pulse rate variability via fingertip PPG assessment has been highly correlated with both time and frequency-domain metrics from ECG-derived HRV indices [10,33]. Although the current study produced significant correlations between ECG- and PPG-derived LnRMSSD values, the relationship fluctuated between recording periods and all the resting comparisons produced “small” yet significant mean differences. The differences in the PPG and ECG correlations found between previous research and the present study may be methodological. Plews et al. [13] investigated the validity of the same smartphone application as in the current study and found near-perfect correlations (*r* = 0.99); however, several differences exist in the procedures, including the ECG equipment set-up, collection procedures, and analysis of the RR intervals. Peng et al. [19] also investigated the extraction of HRV from smartphone PPG signals with five different algorithms and compared them to ECG-derived metrics for 30 healthy participants in the supine position. The obtained RMSSD measurements demonstrated large-to-very-large correlations (*r* = 0.60−0.78); however, all the time-domain parameters displayed insufficient agreement according to the Bland–Altman ratio [19]. The smartphone application implements its unique algorithm to determine RR intervals, which may differ from those used in ECG-derived HRV analysis [13]. It may be that the methodology used to compare ECG and PPG, in addition to the RR interval detection algorithm, significantly impacts HRV metrics, even under resting conditions. 

Though smartphone PPG shows much promise as an alternative to ECG for determining HRV [8], some controversy regarding its efficacy still exists, which may be linked to the physiological differences between PRV and HRV. Previous research has found that PRV seems to overestimate the RMSSD compared to HRV, which was attributed to the difference being due to the pulse transit time or the time needed for a pulse pressure wave to travel to the periphery, commonly the fingers or toes [10,23,34]. Because of the pulse transit time, PRV is heavily influenced by HR, blood pressure changes, and the compliance of the arteries. An increase in HR leads to a temporary increase in blood pressure, reducing arterial compliance, which culminates in increased pulse transit time [22,23]. A significant increase would cause longer interval periods in PRV, which may have caused the trend towards the overestimation of LnRMSSD observed during all the measurement periods of the current study. 

Our findings reflect the results of previous studies with smartphone PPG measurements of LnRMSSD being higher than ECG-derived HRV on average. Though the exact mechanisms behind the possible differences seen in some of the literature between ECG- and PPG-derived HRV metrics have not been fully agreed upon, it was suggested that different positions may cause variations in the accuracy of PRV [10]. The slight deterioration in mean accuracy in seated and standing positions compared to supine may be due to an orthostatic stress-induced increase in sympathetic activation leading to heightened arterial stiffness and decreased pulse wave velocity [15]. While investigating the simultaneous measurements of PPG and ECG, Lu et al. (2009) found good agreement between PRV and HRV in supine and upright positions but saw slight deteriorations with upright posture [35]. In the present study, the participants were instructed to remain motionless during the recordings and all the measurements were taken in the seated position in an attempt to reduce all possible parasympathetic saturation [2,33,36]. Though practical for athletes outside of lab conditions, the seated position may result in increased pulse transit time and, subsequently, worse PRV compared to supine measurements. A recent meta-analysis found a non-significant difference in error among different positions; however, it was concluded that positions are not interchangeable and should be kept consistent for longitudinal monitoring [7]. 

The present findings show that post-exercise simultaneous measurements demonstrated significant differences with moderate ESs and the weakest correlation, but still displayed moderate agreement, though the ratio was the highest compared with pre-exercise measures. Previous research has found that physical activity and mental stimuli are determinants of the inaccuracy of PPG assessment [8,10,14]. This overestimation of HRV from PPG may be linked to the increased sympathetic activity commonly seen during exercise and the physiological effects that follow [37]. It was speculated that the increased differences could be related to changes in the mechanical properties of the arteries, such as an increase in central arterial stiffness due to exercise, which leads to a decrease in pulse wave velocity and decreased PRV, contrasted by a decrease in peripheral arterial stiffness, leading to possible discrepancies between ECG and PPG [37]. Resistance exercise has been associated with an increased HR and breathing rate and spikes in blood pressure [10], as well as transient increases in arterial stiffness for up to 30 min afterwards [38,39]. In the current study, multiple compound exercises were utilized and the Valsalva maneuver was not controlled for, potentially causing the aforementioned sympathetic responses [38,39]. Our results showed significant elevations in HR and reductions in diastolic blood pressure post-exercise; however, no significant changes in systolic pressure from before to after were observed. Additionally, the post-exercise PP was significantly higher than pre-exercise PP, possibly indicating greater turbulent flow and resistance to flow in the smaller peripheral arteries [40]. The combination of these physiological responses may have caused an increased lag in the pulse transit time and inaccurate pulse cycle detection due to artifacts and noise, leading to the weaker correlation and larger ES of the mean difference observed.

The present study also examined the intraday and interday reliability of smartphone PPG measurements under resting conditions. Plews et al. [17] established that multiple HRV measurements, averaged from week to week, were superior to isolated collections taken in a pre–post fashion [17]. Though many studies have analyzed the accuracy of new mobile devices [11,12,13], fewer have investigated reliability through consecutive recordings [21]. The near-perfect intraday reliability can be attributed to the uniformity of the environment in which the recordings took place. Though consecutive measures were taken with a 10 min gap between them, the participants were not heavily stimulated during this time and the environmental conditions (e.g., temperature, humidity, etc.) remained constant. The interday reliability was found to be very large, displaying a slight decrease in comparison to the intraday reliability. This may be due to external factors outside the control of laboratory conditions affecting the participants before the follow-up session. Based on these results, practitioners can expect valid HRV measures on a near-daily basis as long as the conditions during the time of recording remain relatively constant.

### Limitations

Although the study has numerous strengths, the study is not without limitations. The smartphone application used in the current study relies on the camera (i.e., detector) and flash (i.e., light source) systems to acquire PPG data. Therefore, it is worth noting that the quality and recency of the device may affect measurement accuracy. Though the iPhone 6 utilized in this study supported the PPG application and was in good condition, it is several generations old and may account for some proportion of the error observed in the results. The breathing frequency and tidal volume were not controlled; however, this was done to replicate the data collection procedures typically used in field settings. It has also been shown that the breathing rate does not appear to influence the RMSSD, unlike spectral indices [11,20]. The measurements were performed in a well-controlled laboratory setting with researchers overseeing all the procedures to ensure measurement quality, which is not truly indicative of field settings with athletes. Finally, though both males and females were recruited for this study, only 29% of the sample was female. Although there are not physiological mechanisms by which biological sex should impact the relative accuracy of the portable device, the current study did not have adequate statistical power to examine the potential impact of biological sex, as it was beyond the scope of the current study.

## 5. Conclusions

The monitoring of daily HRV outside laboratory conditions with commercially available equipment is a valuable and reliable tool for gauging autonomic modulation. Furthermore, it can provide additional insight into an individual’s recovery status and act as an internal indicator of physiological responses to a bout of exercise or the accumulated effects of training. With the increasing interest in portable devices and wearable technologies, smartphone PPG offers a noninvasive, cost-effective alternative to the traditional ECG method. Though the results of the current study can only be interpreted within the context of an acute bout of physical stress, individuals should avoid taking measurements too soon following exercise or during periods of significant physical strain and increased sympathetic activity, to ensure greater accuracy. However, when taken under resting conditions, smartphone PPG provides valid and time-efficient measurements of HRV.

## Figures and Tables

**Table 1:** Comparison of electrocardiograph (ECG) and photoplethysmography (PPG)-derived log transformation of the root mean square of successive RR differences (LnRMSSD).

		*N*	*M* ± *SD*	*p*	Effect Size		*r*	SEE	Ratio	CE ± 1.96SD	Upper	Lower	Trend (*r*)
Base_Pre1_	ECG	31	4.05 ± 0.65										
	PPG	31	4.38 ± 0.61	0.003	0.42	Small	0.59	0.54	0.13	0.34 ± 1.12	1.46	−0.79	−0.09
Base_Pre2_	ECG	31	4.06 ± 0.63										
	PPG	31	4.29 ± 0.59	0.019	0.30	Small	0.63	0.50	0.12	0.23 ± 1.03	1.26	−0.79	−0.08
RE_Pre1_	ECG	31	4.05 ± 0.59										
	PPG	31	4.25 ± 0.53	0.041	0.26	Small	0.63	0.47	0.11	0.19 ± 0.95	1.15	−0.76	−0.13
RE_Pre2_	ECG	29	3.88 ± 0.62										
	PPG	29	4.15 ± 0.58	0.001	0.36	Small	0.76	0.42	0.10	0.28 ± 0.83	1.07	−0.55	−0.10
RE_Post_	ECG	31	2.44 ± 1.00										
	PPG	31	3.50 ± 0.72	<0.001	1.14	Mod	0.41	0.92	0.16	0.98 ± 0.96	2.94	−0.82	−0.33

Notes: ECG = electrocardiogram; PPG = photoplethysmography; LnRMSSD = log transformation of the root mean square of successive RR differences; *N* = number; *M* ± *SD* = mean ± standard deviation; SEE = standard error of estimate; CE = constant error; Base = baseline; RE = resistance exercise.

**Table 2:** Signal quality category-based comparative analysis.

		*N*	MD	ES	*p*	*r*	*p*	BAR
Optimal	Base_Pre1_	19	0.32	Small	0.058	0.48	0.037	0.08
	Base_Pre2_	20	0.16	Trivial	0.280	0.57	0.009	0.05
	RE_Pre1_	22	0.10	Trivial	0.322	0.56	0.006	0.05
	RE_Pre2_	22	0.09	Trivial	0.051	0.94	<0.001	0.07
	RE_Post_	19	0.66	Moderate	<0.001	0.78	<0.001	0.36
Good	Base_Pre1_	7	0.34	Small	0.009	0.92	0.003	0.07
	Base_Pre2_	9	0.32	Small	0.001	0.94	<0.001	0.05
	RE_Pre1_	7	0.36	Small	0.058	0.92	0.003	0.04
	RE_Pre2_	4	0.80	Moderate	0.039	0.92	0.083	0.08
	RE_Post_	6	1.52	Large	<0.001	−0.90	0.015	0.37
Failed	Base_Pre1_	5	0.46	Small	0.066	0.26	0.670	0.07
	Base_Pre2_	2	0.55	Small	0.272	1.00	<0.001	0.05
	RE_Pre1_	2	0.75	Moderate	0.403	−1.00	<0.001	0.04
	RE_Pre2_	3	0.93	Moderate	0.088	−0.80	0.401	0.06
	RE_Post_	6	1.73	Large	0.036	−0.28	0.596	0.30

Notes: MD = mean difference; ES = effect size; BAR = Bland–Altman ratio; Base = baseline; RE = resistance exercise.

**Table 3:** Intraday and interday reliability of PPG-derived LnRMSSD measurements.

		*M* ± *SD*	*α*	*χ^2^*	*p*	ICC	95% CI		*p*
							Lower	Upper	
LnRMSSD	Intraday	4.34 ± 0.20	0.91	2.08	0.149	0.91	0.812	0.956	<0.001
	Interday	4.28 ± 0.29	0.87	5.02	0.171	0.88	0.795	0.940	<0.001

Notes: PPG = photoplethysmography; LnRMSSD = log transformation of the root mean square of successive RR differences; M ± SD = mean ± standard deviation; α = Cronbach’s alpha; ICC = intra-class correlations; CI = confidence intervals.
